# Fast Dissolving Resveratrol–Polyvinylpyrrolidone Nanofibrous Films Fabricated in Bulk Using a Special Hole Electrospinning Technique

**DOI:** 10.3390/polym18070795

**Published:** 2026-03-25

**Authors:** Qiuyun Guo, He Lv, Ran Dong, Wei Yi, Hongxi Wang, Hui Liu, Deng-Guang Yu, Tao Yi

**Affiliations:** 1 School of Materials and Chemistry, University of Shanghai for Science and Technology, Shanghai 200093, China; 233393129@st.usst.edu.cn (Q.G.); 251620330@st.usst.edu.cn (R.D.); huiliu@usst.edu.cn (H.L.); 2 Department of Chemistry, Tonghua Normal University, Tonghua 134002, China; 2024060@thnu.edu.cn; 3 FORYOU Mechatronics (Shanghai) Ltd., Shanghai 201114, China; lily@ablpropharma.com (W.Y.); wang@i-foryou.com.cn (H.W.); 4 Faculty of Health Sciences and Sports, Macao Polytechnic University, Macau 999078, China

**Keywords:** large-scale productions, hole electrospinning, polyvinylpyrrolidone, nanofibers, single-needle electrospinning, free surface electrospinning, resveratrol

## Abstract

Batch production of nanomaterials is highly desired for developing commercial nanoproducts. Here, a brand-new electrospinning method, termed hole electrospinning, was developed for batch production of drug-loaded polymeric nanofibers. Using resveratrol and polyvinylpyrrolidone as model drug and filament-forming matrix, respectively, both hole and single-needle electrospinning were conducted. The resultant nanofibrous films were compared in terms of morphology, physical and thermal properties, mechanical performance, fast-dissolution rate, and antioxidant activity. Analytical and characterization results verified that nanofibers from different processes showed no significant differences in morphology, diameter, porosity, tensile strength, amorphous state, fast-dissolution performance and antioxidant activity. However, hole electrospinning provided 13.3-fold higher productivity than single-needle electrospinning, better drug encapsulation efficiency (97.3 ± 4.5% versus 83.7 ± 6.1%), and higher energy efficiency (0.0393 W/g versus 0.1247 W/g). Based on the protocols reported here, not only was a batch nano-conversion method for polymeric engineering developed, but also an attractive approach for the large-scale production of various complex configurations was proposed for potential commercial nanostructure-based products.

## 1. Introduction

Active biomolecules are highly essential for human health and well-being. However, most of them are water-insoluble, which greatly limits their potential applications [1,2,3]. Many material conversion methods have been introduced to resolve this issue. Particularly in this nano-era, plenty of nanotechniques have been employed to produce active biomolecule-loaded nanoproducts [4,5,6]. In addition to particulate nanofabrication methods such as co-precipitation and electrospraying, fibrous nano-preparation methods, represented by electrospinning, are also popular due to their many special advantages [7,8,9,10].

The large-scale production of nanoproducts for potential commercial applications poses one of the most important challenges to researchers [11]. This is not only the ultimate goal of laboratory research but also a requirement for the sustainable development of our society [12,13,14]. Among the transitions of various nanoproducts from laboratory to factory, batch productions of complex nanostructures and devices represent the highest peak—one that seems insurmountable. Although there are abundant reports on “top-down” and “bottom-up” methods for creating complicated nanostructures in laboratories worldwide [15,16,17], almost no commercial products have emerged from these efforts. This sharp contrast stems precisely from the difficulty of large-scale preparation of complex nanostructures and devices in a simple and robust manner.

Initially, the popularity of electrospinning can be attributed to two aspects. One aspect is the simple, convenient, and cost-effective production of polymeric nanofibers, which hold great promise for large-scale production, as has been demonstrated by some commercial nanofiber-based products [18,19,20,21]. The other aspect is the unique properties of electrospun nanofibrous mats, including well-recognized characteristics such as large surface area, small diameters, high porosity, and a non-woven structural pattern resembling the extracellular matrix [22,23,24,25,26,27]. Following the progress in nanoscience and nanotechnology, electrospinning is now evolving from traditional single-fluid blending electrospinning and the resultant monolithic nanofibers to multiple-fluid electrospinning and related nanofibers with complex configurations [28,29,30,31]. Besides the popular coaxial electrospinning and its core-sheath nanofibers, which are widely exploited to encapsulate active ingredients, create polymer/drug gradient distributions, exert active or blank coatings on nanofibers, and serve as templates for manipulating molecular self-assembly [32,33,34,35,36,37,38,39], side-by-side electrospinning and the resultant Janus nanofibers are finding applications in various scientific disciplines for building process-structure-function-performance relationships [40,41,42,43]. Moreover, even more complicated multiple-chamber nanostructures such as triaxial, tri-section Janus, and integrated combinations of core-sheath and Janus structures have been successively reported in the literature [44,45]. Although these complex nanostructures have been successfully demonstrated to be useful for targeted applications, their large-scale production remains unresolved, except for several reports on batch production of bi-chamber core-sheath nanofibers [46,47,48].

Based on the introduction of working fluids into the electrical field, electrospinning can be divided into two categories. One is needle-based electrospinning, which represents the initial case of this technique and is closely related to the effect that electric charges tend to accumulate on the metal tip to form a Taylor cone followed by a straight fluid jet and subsequent bending and whipping in unstable drawing regions [49,50,51,52,53]. The other is free surface electrospinning [54,55,56,57,58]. The former has the advantage of facilitating the creation of complex nanostructures from the spinneret’s nozzle, which acts as a structural template, but has the disadvantage of difficult scale-up. The latter, just the opposite, has the advantage of large-scale production, but has the disadvantage of being very difficult to manipulate in terms of the complex nanostructures and configurations of the final nanofibers. Here, based on these two divisions, we hypothesize a third division, which can be viewed as an integration of needle-based electrospinning and free surface electrospinning for batch production of nanofibers. Meanwhile, two modifications were made: first, the needle was replaced by a solid copper line [59]; second, the free surface was confined to a region within a hole at the bottom of the tank holding the working fluid.

Resveratrol (Res) is a natural polyphenolic compound [60]. Resveratrol and its derivatives are mainly found in at least 72 plant species belonging to 21 families and 31 genera, including Vitis (grape), Polygonum, and Arachis (peanut). These plants include common medicinal species such as Polygonum cuspidatum, Cassia, and mulberry, as well as crops such as grapes and peanuts. Current research suggests that Res may be beneficial for cardiovascular health, neuroprotection, and metabolic regulation. Its antioxidant mechanism involves eliminating free radicals and reducing oxidative stress damage; its anti-inflammatory mechanism involves inhibiting inflammatory factors (such as the NF-κB pathway); and its longevity-related mechanism involves activating the SIRT1 protein to delay cellular aging. However, these beneficial effects are limited by its poor water solubility [61,62,63,64]. Polyvinylpyrrolidone (PVP) is a widely used hydrophilic polymer that has been reported to enhance the solubility of many poorly soluble molecules and exhibits excellent fiber-forming and electrospinnable properties in various solvents [65,66,67]. Meanwhile, electrospun PVP nanofibers are easy to endow with the desired antioxidant property for various desired applications, such as wound dressings and food packaging [68,69,70].

**Figure 1:**
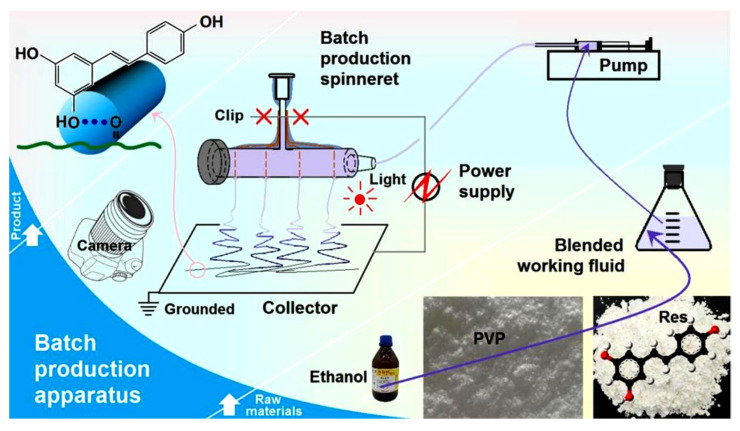
A diagram showing the conversions of PVP and Res through a hole electrospinning process for batch production of nanofibrous drug–polymer composites.

Herein, a batch electrospinning process, characterized by an array of holes for guiding the working fluid (and thus termed “hole electrospinning”), was developed to encapsulate Res into PVP nanofibers, forming a novel hydrophilic nanocomposite (a schematic diagram is shown in Figure 1). These composites enable rapid dissolution of Res upon contact with water. Single-fluid needle electrospinning was also conducted, using the same working fluid for comparison, to verify the effectiveness of batch production via hole electrospinning.

## 2. Materials and Methods

### 2.1. Materials

Resveratrol (Res) with a purity of 98% was obtained from Shanghai Haosheng Biomed. Co., Ltd. (Shanghai, China). Polyvinylpyrrolidone (PVP) K60 (Mw = 360,000 g/mol), 2,2-diphenyl-1-picrylhydrazyl (DPPH) and analytical reagent anhydrous ethanol were purchased from Sigma-Aldrich (Shanghai, China). Phosphate buffer solution (PBS, pH 7.0, 0.1 M) was obtained from Tianjin Zhiyuan Chemical Reagent Co., Ltd. (Tianjin, China). Double-distilled water was used throughout this study. 

### 2.2. Electrospinning

A home-made assembled spinneret and the corresponding electrospinning apparatus was exploited for batch production of medicated nanofibers. The monoaxial needle spinneret was utilized to conduct single-fluid electrospinning to prepare medicated nanofibers as control. Two syringe pumps were exploited to organize the whole electrospinning apparatus: one was KDS200 (Cole-Parmer, Vernon Hills, IL, USA) for fixation of the spinneret, and the other was KDS100 (Cole-Parmer, Vernon Hills, IL, USA) for transferring the working fluid. The electrostatic high voltage was generated from a power supply (ZGF60 kV/2 mA, Wuhan Hua-Tian High Power Co., Ltd., Wuhan, China). The collector consisted of hard cardboard covered with aluminum foil. The collector was fixed at a constant distance of 20 cm from the spinneret tip. The flow rate and applied voltage for needle electrospinning were 3 mL/h and 18.7 kV, respectively, whereas for hole electrospinning, these values were 40 mL/h and 26.2 kV, respectively. These working parameters were determined through direct observation using a camera (Canon G7X, Tokyo, Japan) to ensure a continuous and robust working process. The air conditioning was turned off to prevent potential interference. The ambient temperature and relative humidity were maintained at 10.1 ± 1.8 °C and 39 ± 4%, respectively.

The working fluid was prepared by adding 7.5 g of Res to 500 mL of anhydrous ethanol. After complete dissolution, 42.5 g of PVP was added with stirring. The resultant solution was transparent and pale yellow. The viscosity, conductivity, and surface tension of ethanol, pure PVP solution (containing 4.25 g PVP in 50 mL ethanol) and PVP-Res working fluid were measured at 10 °C. The viscosity of polymer solutions was determined by a rotation viscometer (NDJ-7, Shanghai, China). Conductivity was measured using the electrode method with a resistivity meter (Leici DDS-307, Shanghai, China). Surface tension was measured using the hanging drop method with a contact angle/surface tension meter (Dataphysics DCAT21, Göttingen, Germany).

### 2.3. Morphologies of the Nanofibers from Various Electrospinning Processes

The nanofibers fabricated by hole electrospinning and conventional needle electrospinning were examined using a scanning electron microscope (SEM; FEI Quanta 450 FEG, FEI Corporation, Hillsboro, OR, USA). Prior to SEM examination, the nanofibrous films were gold-coated for 90 s. The nanofiber diameters were measured using ImageJ software V1.8.0 (NIH, Bethesda, MD, USA), while Origin 6.1 was used for diameter distribution statistics.

### 2.4. Physical and Chemical Properties Characterizations

The physical states of raw components and their composite nanofibers from various electrospinning processes were evaluated by X-ray diffraction (XRD) using a Bruker D8 Advance diffractometer (Bruker, Bremen, Germany) equipped with a Cu Kα source (40 kV, 40 mA), at a scanning rate of 10°/min and a step size of 0.02°.

Thermal properties (TG-DSC) of PVP K60, resveratrol powder, needle electrospun fibers, and hole electrospun nanofibers were tested using a simultaneous thermal analyzer (Netzsch STA 449, NETZSCH, Selb, Germany). An amount of about 4.0 to 5.0 mg sample was evenly placed in an alumina crucible and heated at a rate of 10 °C/min from 30 °C to 800 °C under a nitrogen atmosphere at a flow rate of 50 mL/min, while recording weight loss and heat flow data.

Tensile strength (TS) and elongation at break (EB) were determined using a Precisionline vario tensile testing machine (Zwick Co., Ltd., Ulm, Germany). Prior to testing, fiber membrane thickness was measured with a micrometer, and mechanical property evaluation was conducted at a tensile speed of 10 mm min^−1^.

Determination of the porosity (P) of a fiber membrane is achieved using the liquid impregnation method, as reported in the literature [71]. The fiber membrane is cut into a regular shape with neat edges. Using a vernier caliper, measure the length (L), width (W), and thickness (H), then calculate the apparent volume, denoted as V1. Place the sample in an oven and dry at 40–50 °C for 2 h. Remove and cool to room temperature in a desiccator. Weigh the dry mass using a precision balance, denoted as W1. Immerse the sample completely in isopropyl alcohol for 2 h, then remove from the isopropyl alcohol. Absorb excess surface droplets with filter paper and promptly weigh the wet mass, denoted as W_2_. Calculate the absorbed volume of isopropyl alcohol, denoted as V_2_. All measurements are repeated three times. V_1_, V_2_, and the porosity value (P) are calculated using the following formula:

(1) $$V_{1}=L\times W\times H$$

(2) $$V_{2}=\frac{W_{2}-W_{1}}{\rho }$$

(3) $$P=\frac{V_{1}}{V_{2}}\times 100\%$$

In this formula, ρ represents the density of isopropanol, taken as 0.785 g/cm^3^. If the test temperature differs, apply a correction using the following formula:

(4) $$\rho_{T}=\rho_{25}-0.00085\times \left(T-25\right)$$

### 2.5. DPPH Antioxidant Activity

To evaluate the antioxidant activity of the nanofiber membranes, DPPH radical scavenging assays were conducted. A fresh 0.1 mM DPPH solution in ethanol was prepared. The nanofiber membranes (25 mg) were immersed in 5 mL of the DPPH solution and incubated under dark conditions at 25 °C for 30 min. Absorbance at 517 nm was recorded using a UV–vis spectrophotometer (UV-2102PC, Unico Instrument Co., Ltd., Shanghai, China), and absorbance changes were used to assess the radical scavenging activity before and after sample immersion (*n* = 3).

### 2.6. Observations of Fast-Dissolution Processes

A glass slide was placed on the collector to collect the electrospun nanofibers. A drop of water was dripped onto the electrospun nanofibrous mat. An optical microscope was used to record the fast-dissolution process. After the water droplet had dried, the boundaries of the solid nanofibers, as well as the traces of dried sections, were captured by a camera (Canon G7X, Tokyo, Japan).

### 2.7. Drug Encapsulation Efficiency (EE%) and Drug Loading (DL%)

The UV–vis spectrophotometer was used to measure the absorbance of Res in aqueous solution at 295 nm. A calibration curve was established between absorbance (A) and Res concentration (C, µg/mL) to calculate the concentration from the measured A value.

Res was recovered from nanofibers prepared by batch-hole electrospinning and needle electrospinning. A fibrous film of weight W_f_ was dissolved in anhydrous ethanol. Then, 1.0 mL of the ethanolic solution was gradually added to 1.0 L of distilled water under stirring to release Res. The drug mass in the fibrous film (W_d_) was then calculated from the concentration determined by UV–vis spectrophotometry. Thus, DL% was calculated according to Equation (5):

DL% = (Wd/Wf) × 100(5)

EE% was calculated according to Equation (6):

EE% = (W_d_/W_p_) × 100 (6)

where W_p_ is the mass of Res initially added to the working fluid.

### 2.8. In Vitro Dissolution Tests

The release profiles of Res from batch-produced nanofibers, needle-electrospun nanofibers, and raw Res particles were evaluated in vitro. Samples containing 20 mg of Res were immersed in the vessels of a water-bath constant-temperature shaker, which was kept at 37 °C and shaken at 50 rpm. The vessels contained 600 mL of PBS (pH 7.0, 0.1 M). 5.0-mL aliquots were drawn from the dissolution vessels at predetermined time intervals. Meanwhile, the same volume of fresh PBS was added to the vessels to maintain a constant total dissolution volume. The cumulative release percentage of Res (Q, %) was calculated using the following Equation (7):

(7) $$Q(\%)=\frac{C_{n}\times V_{0}+\sum_{i=1}^{n-1}C_{i}\times V}{A_{0}}\times 100$$

where V_0_ is the volume of the dissolution medium (600 mL), V is the volume of the drawn sample (5.0 mL), A_0_ is the theoretical drug amount in each sample (mg), C is the Res concentration measured in the nth aliquot (mg/L), and Cᵢ is the Res concentration in the ith aliquot (mg/L).

### 2.9. Statistical Analysis

All experiments were performed at least in triplicate throughout this study. Data analysis and graph drawing were performed using Origin 6.0 software. The data were presented as mean ± standard deviation (SD). Significant differences between groups were analyzed using two-tailed paired or unpaired Student’s *t*-tests and a one-way analysis of variance (ANOVA), and a value of *p* < 0.05 was considered statistically significant.

## 3. Results and Discussion

### 3.1. Batch Productions of Nanofibrous Composites

A series of parameters influences a continuous and robust electrospinning process and the quality of the resultant nanofibers. These parameters can be categorized into three groups [72,73,74]: (1) the properties of the working fluid for easy filament formation, such as viscosity, surface tension, and conductivity; (2) operational parameters, including fluid flow rate, applied voltage, collection distance, and temperature of the working fluid; and (3) environmental parameters, mainly ambient temperature and humidity. Among all these parameters, a reasonable matching of the fluid flow rate and the applied voltage is a key factor, besides the fluid’s properties. Here, the parameters of both needle and hole electrospinning were optimized by directly observing the working process using a smartphone and the Canon camera, judged by a stable Taylor cone without fluid dripping. The viscosity, surface tension, and conductivity of the ethanol, pure PVP solution, and Res-loaded PVP solution were included in Table 1. The addition of Res in the PVP solution increased the viscosity, slightly increased the surface tension, but slightly decreased the conductivity. These variations had very limited influences on the electrospinnability of the Res and PVP blended solution. To avoid the negative influence of the air conditioner mounted on the room ceiling, it was turned off; consequently, the ambient temperature and relative humidity were 10.3 °C and 40%, respectively, matching the outdoor conditions.

**Figure 2:**
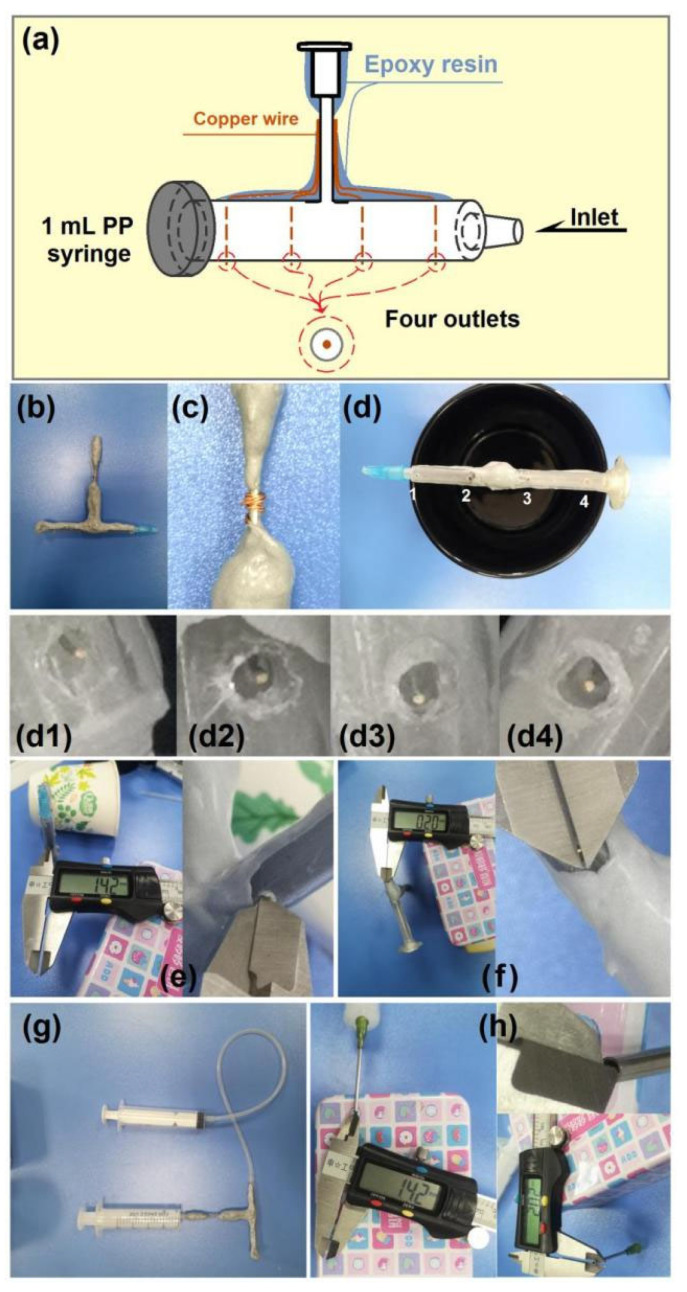
Details of the brand-new spinneret: (**a**) diagram of the spinneret preparation details; (**b**) digital image of the whole spinneret; (**c**) exposed site for transferring high electrostatic energy; (**d**) digital image of the four-hole array at the bottom of the syringe ((**d1**–**d4**): images of the four outlets); (**e**) outlet hole with a diameter of ~1.42 mm; (**f**) copper wire with a diameter of ~0.20 mm; (**g**) connection of the spinneret with two syringes—one for fixing the spinneret, the other for holding the working fluid; (**h**) monoaxial metal capillary (inner diameter: 1.42 mm; outer diameter: 2.02 mm) used for preparing nanofibers for comparison.

Batch fabrication of nanoproducts is highly pursued. Needle-based electrospinning requires many mono-axial metal capillaries to elevate yields. Thus, free surface electrospinning is regarded as a more straightforward method for the large-scale production of nanofibers. Regardless of the electrospinning type, the spinneret is regarded as the most important and innovative element among the parts of an electrospinning system [75,76,77]. Here, a special assembled spinneret was developed for batch production of nanofibers. Details about this brand-new spinneret are included in Figure 2.

A diagram of the inner structure and construction details of the spinneret is shown in Figure 2a. The spinneret contained a polypropylene (PP) syringe with 1 mL volume as the reservoir for working fluids, a needle for easy fixation of the whole spinneret, an inlet at the side, and four outlets at the bottom of the syringe. Four copper wires were gathered from the centers of the holes to the upright metal capillaries. These copper wires were coated with epoxy resin. A complete digital image of the spinneret is exhibited in Figure 2b. The spinneret weighs 5.7 g, making it very light for easy implementation. The assembled spinneret has only a very short naked section, which was used to transfer high electrostatic energy (Figure 2c). There are four holes at the bottom of the syringe, as shown in Figure 2d. In the future, yields can be further increased simply by increasing the number of holes. Digital images of the four hole outlets at the bottom of the syringe, i.e., d1 to d4, are included in Figure 2(d1–d4). As shown in Figure 2e, the outlet hole has a diameter of approximately 1.42 mm, whereas the copper wire has a diameter of approximately 0.20 mm (Figure 2f). The spinneret can be connected to two syringes: one vertical syringe for easy fixation of the whole spinneret, and the other used for conveying the working fluid through highly elastic silicon tubing (Figure 2g). For comparison, a mono-axial metal capillary with inner and outer diameters of 1.42 mm and 2.02 mm (Figure 2h), respectively, was used for preparing nanofibers through a traditional blending electrospinning procedure.

**Figure 3:**
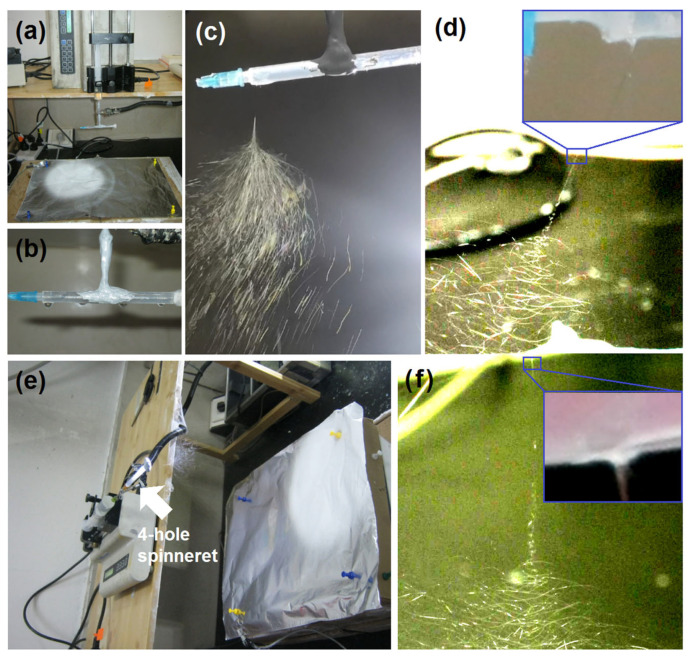
Some typical failed batch production processes: (**a**) a digital image of the electrospinning process through a spinneret with four holes but no copper wires; (**b**) four droplets from the four holes when no voltage is applied; (**c**) a digital image of electrospinning, which occurred at only one hole (taken using a smartphone); (**d**) a typical straight fluid jet, followed by gradually increased bending and whipping, taken by a camera, with the top-right inset showing the small Taylor cone; (**e**) the fluid was introduced from the side during multi-hole electrospinning; (**f**) a typical straight fluid jet, followed by gradually increased bending and whipping with a small Taylor cone (the top-right inset), taken by a camera.

Initially, the assembled spinneret contained no copper wires, a condition that frequently led to process failures (Figure 3). Figure 3a presents a digital image of the electrospinning process using a four-hole spinneret without copper wires. Although four distinct droplets were observed at the four holes in the absence of applied voltage (Figure 3b), electrospinning occurred at only one hole upon voltage application. The remaining holes appeared to be sealed due to increased surface tension under conductive conditions. This elevation in surface tension can be attributed to several mechanisms: (1) charge repulsion effect: the accumulation of free charges (ions or electrons) on the liquid surface generates electrostatic repulsion between like charges, which resists surface contraction and thereby increases surface tension; and (2) polarization and interfacial energy changes: external electric fields or spontaneous polarization (such as the orientation of polar molecules) can modify the interfacial energy of the liquid. In conductive substances, charge redistribution induced by the electric field enhances surface molecular polarity, necessitating greater energy to maintain a stable interface. Certainly, the one-hole electrospinning can still run smoothly and continuously, as indicated by Figure 3c,d. Although the taken spinning “phenomena” are different, they represent the same working processes. The syringe had a diameter of 6.8 mm; thus, the straight fluid jet was estimated to be 11.3 mm, whereas the Taylor cone was very small, as indicated in the top-right inset of Figure 3d.

Interestingly, a similar phenomenon was observed when the fluid was introduced from the side for hole electrospinning (Figure 3e). Despite the formation of a typical straight fluid jet followed by progressive bending and whipping motions (Figure 3f) with a very small Taylor cone (as indicated by its up-right inset), electrospinning still occurred exclusively at one of the bottom holes. To mitigate this surface sealing effect under high voltage, copper wires were inserted through the four bottom holes to realize the batch production effect.

**Figure 4:**
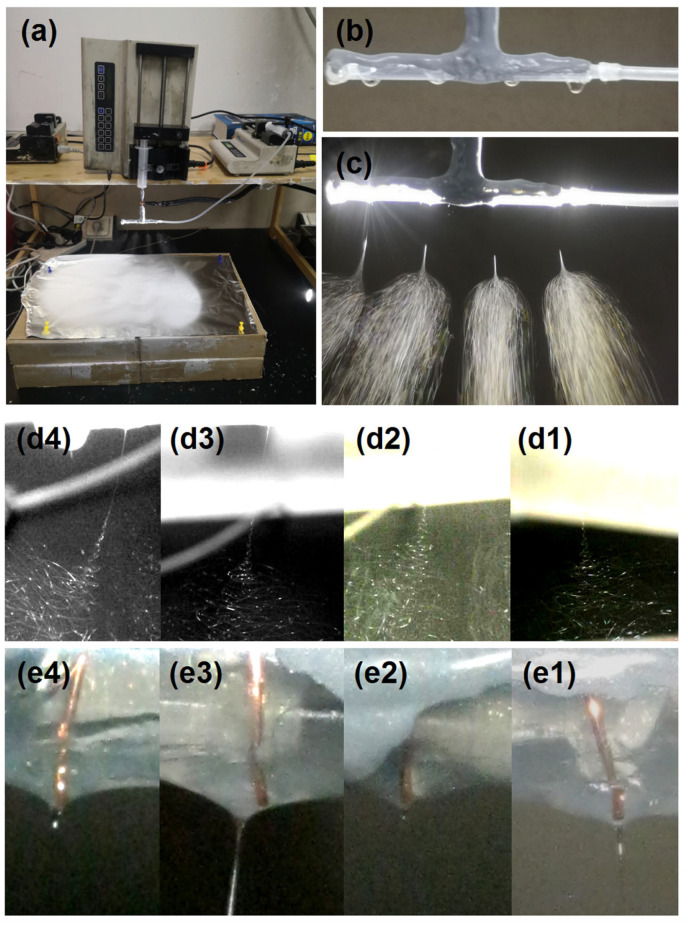
Batch production of nanofibers via multi-hole electrospinning: (**a**) photograph of the batch electrospinning setup; (**b**) simultaneous extrusion of four droplets from the four-hole spinneret in the absence of applied voltage; (**c**) operational states of the four bottom nozzles during electrospinning; (**d1**–**d4**) a typical four-stage electrospinning process showing the evolution from Taylor cone formation, through straight fluid jet ejection, to unstable bending and whipping motions; (**e1**–**e4**) corresponding Taylor cones formed at the four holes with central copper-wire electrodes.

As shown in Figure 4, the homemade batch production apparatus is presented, along with details of the working processes. Similar to traditional needle electrospinning, the electrospinning apparatus consists of a novel spinneret, two pumps (a KDS200 for fixation and a KDS100 for quantitative delivery of working fluid), a power supply (not shown, but indicated by the alligator clip connected to the assembled spinneret in Figure 4a, and a collector (a whole electrospinning apparatus and the working parameters are included in the Supplementary Video S1). At a fluid flow rate of 40 mL/h, droplets of working fluid were continuously pumped out, as shown in the digital image in Figure 4b. When the applied voltage was increased to 14 kV, the electrospinning process was initiated at one of the holes, but was unstable and did not involve all holes. When the applied voltage was adjusted to 26.2 kV, a robust and continuous electrospinning process was achieved with all four holes active. A digital photograph is provided in Figure 4c. These processes exhibited similarly sized Taylor cones, straight fluid jets of comparable length, and similar apparent “division” phenomena (a working process is provided in the Supplementary Video S2).

However, the “division” is an artifact resulting from the Smartphone P60 (Huawei, Shenzhen, China) capture speed being lower than the moving speed of the fluid jets. When a camera (Canon G7X, Tokyo, Japan) was used to retake the photograph, all four holes exhibited the typical three stages of operation—the Taylor cone, straight fluid jet, and bending and whipping unstable regions—as shown in Figure 4(d1–d4), corresponding to holes 1–4, respectively. Correspondingly, their Taylor cones are shown in Figure 4(e1–e4), respectively. The copper wires are clearly visible in the working fluid, conveying electrostatic energy to the tips to overcome the surface tension barrier, initiating the Taylor cone and subsequent nanofibrous fabrication procedures.

**Figure 5:**
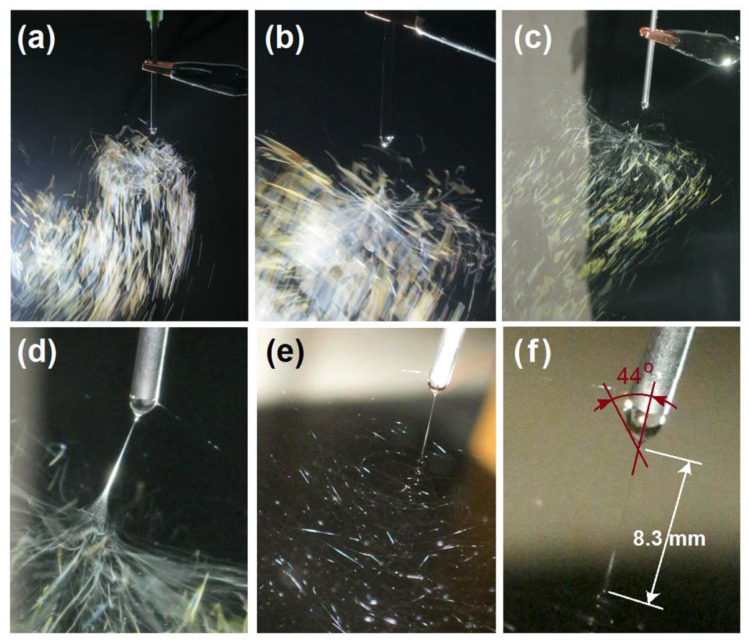
Special phenomena observed in needle-based single-fluid electrospinning: (**a**–**d**) digital images showing the electrospinning process from various angles with a metal capillary spinneret; (**e**) digital image of the operating process recorded using a Canon camera; and (**f**) magnified view of the Taylor cone and the straight fluid jet.

Just as reported in many publications, the single-needle electrospinning showed a continuous “ejection and splitting” procedure, as shown in Figure 5a–d, which were taken around the spinneret tip from various angles. It is interesting that these “divisions” appear colorful due to the interactions between the fluid jets and the camera flash. Similar to the batch production processes, the real working process still involves the typical three successive steps: the Taylor cone, the straight fluid jet, and bending and whipping instabilities with a gradual increase in loop diameter, as shown in Figure 5e. An enlarged image of the Taylor cone and the straight fluid jet is also given in Figure 5f, from which it can be estimated that the Taylor cone has a half-angle of 44° and a straight fluid jet length of 8.3 mm. Compared with the batch production processes, the Taylor cone is larger, but the straight fluid jet is longer due to the higher applied voltage of 26.2 kV.

Based on the formula for electric power consumption (P, in watts [W]), which equals the product of applied voltage (U, in volts [V]) and generated current (I, in amperes [A]), i.e., P = U × I, the batch process had a power consumption of [(26.2 × 1000) × (0.006/1000)] = 0.1572 W. The traditional needle process had a value of [(18.7 × 1000) × (0.002/1000)] = 0.0374 W. However, the former treated 40 mL of working fluid every hour, which produced [40 × (8.5% + 1.5%)] = 4.0 g in theory. The latter treated 3 mL of the working fluid each hour, creating [3 × (8.5% + 1.5%)] = 0.3 g of solid nanofibers in theory. Thus, the specific energy consumption of the batch production and traditional needle electrospinning are 0.1572/4.0 = 0.0393 W/g and 0.0374/0.3 = 0.1247 W/g, respectively. In other words, the batch production achieves an energy efficiency of (0.1247/0.0393) × 100% = 317.3% that of the traditional process, greatly saving energy, which is very important for the commercial production of electrospun nanofibers.

### 3.2. Uniformity of the Nanofibers from Different Holes

**Figure 6:**
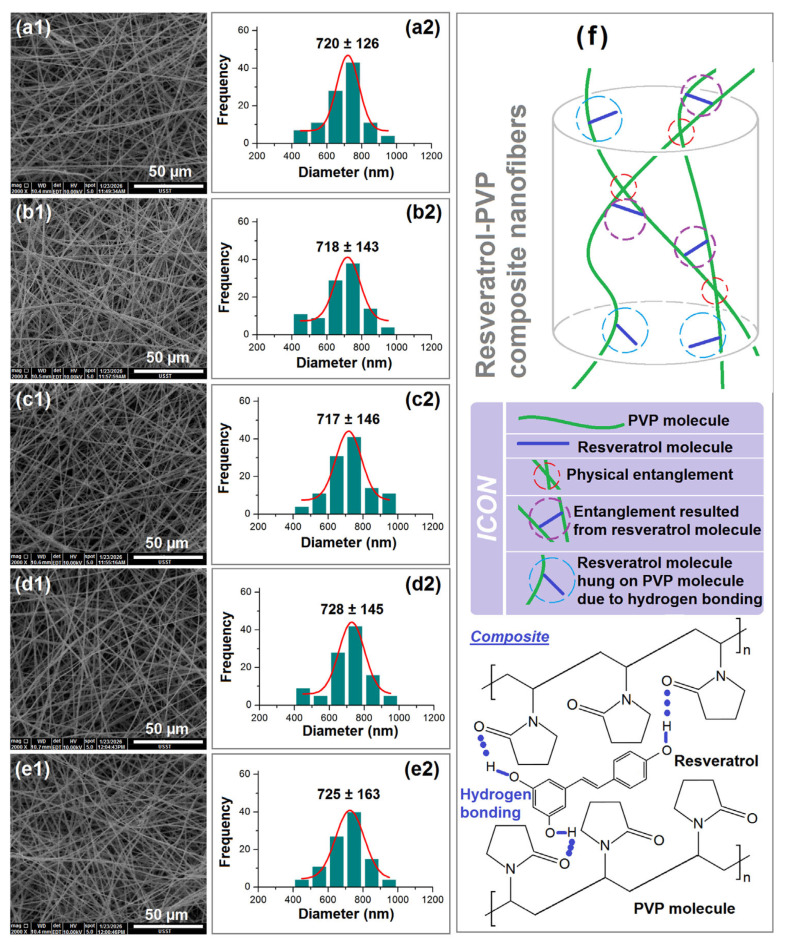
SEM images of nanofibers prepared by hole electrospinning (**a1**–**d2**) and single-fluid needle electrospinning (**e1**), along with their diameter distributions (images (**a2**–**d2**) and (**e2**)). (**f**) A diagram showing the molecular interactions between PVP and Res molecules for forming their composite nanofibers.

SEM images of the nanofibers from the hole batch electrospinning and the single-fluid needle electrospinning, as well as their diameter distributions, are included in Figure 6. Four samples were collected from the collector of batch production, corresponding to the positions of the four holes. As indicated in Figure 6(a1–d1), the fibers from holes 1, 2, 3, and 4 display similar straight linear morphologies with very few beads or spindles. This is due to the excellent electrospinnability of the Res-PVP co-blended working fluid. Using a horizontal collector instead of the present static collector would effectively mix the nanofibers from the four holes, making the collected nanofibrous mats even more homogeneous. As estimated by ImageJ, the nanofibers from holes 1 to 4 have average diameters of 720 ± 126, 718 ± 143, 717 ± 146, and 728 ± 145 nm, as shown in Figure 6(a2–d2), respectively. They exhibit no significant differences, as anticipated.

Figure 6(e1,e2) show the morphology of nanofibers from single-fluid needle-based electrospinning and their size distributions, respectively. They had a straight linear morphology, with an average diameter of 725 ± 163 nm. Still, there were no significant differences between the nanofibers from the batch production processes and those from single-needle electrospinning. In theory, an increase in treated working fluid would increase the diameters of nanofibers, whereas an increase in applied voltage would further reduce the nanofibers’ diameters. These two effects mutually offset each other to result in nanofibers with similar diameters and diameter distributions.

As indicated in their molecular formulas in Figure 6f, one Res molecule has three -OH groups, whereas there are many -C=O groups in PVP molecules. This means that they are highly compatible due to abundant hydrogen bonding. The hydrogen bonds not only make Res molecules attach to PVP molecules, but also result in new entanglements between two PVP molecules, besides the traditional physical entanglements. The hydrogen bonding, together with the hydrophobic interactions between the two benzene rings of Res and the carbon skeleton of PVP, would be favorable for providing high drug loading capacity and EE% during the working process.

### 3.3. Comparisons of Physical State and Properties of the Nanofibers from the Batch Production Processes and Also the Single-Needle Production

**Figure 7:**
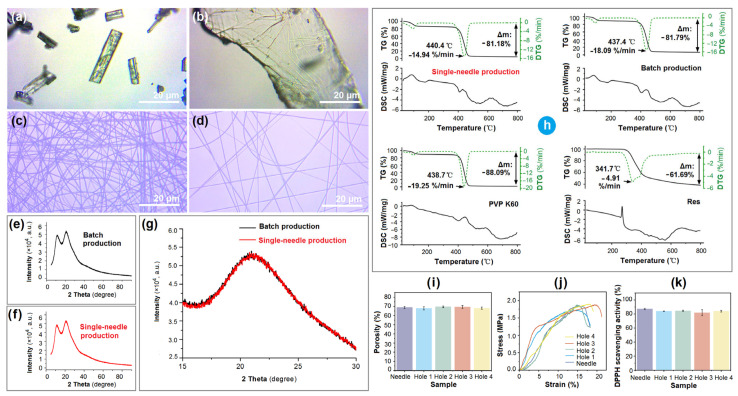
Comparisons of the physical and chemical properties of nanofibrous films created using various electrospinning techniques: (**a**) OM images of raw Res particles; (**b**) OM images of raw PVP powders; (**c**) OM images of nanofibers from batch production; (**d**) OM images of nanofibers from single-needle electrospinning; (**e**) XRD patterns of nanofibers from batch production; (**f**) XRD patterns of nanofibers from single-needle electrospinning; (**g**) direct comparison between the two electrospun nanofibers in the 2θ range of 15–30°, showing no sharp peaks characteristic of raw Res particles; (**h**) thermal properties of PVP, Res, and their nanofibers from single-needle and batch electrospinning processes; and (**i**) porosity, (**j**) stress, and (**k**) 2,2-diphenyl-1-picrylhydrazyl (DPPH) scavenging activity of the nanofibers produced using single-needle electrospinning and batch processes, respectively (Hole 1 to Hole 4 refer to nanofibers collected under the corresponding holes).

Raw Res particles are mainly prism-shaped (Figure 7a). The particles exhibit a colorful appearance, displaying red, blue, green, and purple hues due to the spectral effect of the crystal surface. In contrast, the PVP powders have a larger volume and are colorless (Figure 7b), suggesting an amorphous physical state. Similarly, their nanofibrous products, whether from batch production (Figure 7c) or single-needle electrospinning (Figure 7d), show no color in their optical microscopic (OM) images, indicating that these are similarly amorphous fibrous nanocomposites.

XRD patterns of nanofibers from batch production and those from single-needle electrospinning are shown in Figure 7e,f, respectively. Not only are their patterns almost identical, but their background noise is also highly similar. This is easy to understand: although produced by different electrospinning processes, they are the same fibrous nanocomposites, with almost identical components and compositions. Meanwhile, XRD has a detection sensitivity of about 4% [43]. Thus, subtle differences are difficult to discern, particularly when a large y-axis range is applied to both the drug and polymeric composites, which can easily mislead readers and raise concerns.

The Raw Res XRD patterns exhibit several sharp peaks between 15° and 30° [33]. After Res molecules are encapsulated into PVP nanofibers through electrospinning, regardless of the production method, they lose their characteristic sharp peaks in the composite patterns. A direct comparison of the two kinds of electrospun nanofibers within the 2θ range of 15° to 30° is shown in Figure 7g; no sharp peaks characteristic of raw Res are present.

The thermal properties of PVP, Res, and their nanofibers from single-needle and batch electrospinning processes are included in Figure 7h. Apparently, the drug-loaded nanofibers from the needle and hole electrospinning had very similar thermal behaviors. The measured porosity of the single-fluid electrospun nanofibers was 68.78 ± 1.23. The average values for the nanofibers under the four holes were 68.68 ± 1.36, respectively. These results suggested that the porosity of single-needle electrospinning and hole electrospinning showed no significant differences, and the nanofibers under the four holes were uniform (Figure 7i). The mechanical performances of the nanofibers are shown in Figure 7j, the single-needle nanofibers and those nanofibers under the four holes are highly similar, except those under hole 3 had a longest strain. As for the antioxidant activity, these Res-loaded nanofibers, regardless of whether they were produced by hole electrospinning or needle electrospinning, and regardless of various holes, had similar scavenging activity, as indicated in Figure 7k. By the way, in this investigation, the Res-loaded PVP nanofibrous films were utilized to demonstrate the usefulness of hole electrospinning for batch production of electrospun nanofibers. As for their targeted functional applications, they can be further treated for applications where anti-oxidation is needed, such as wound dressings and food active packaging [78].

### 3.4. Fast-Dissolution Performance of the Res from Nanofibrous Films Resulted from Different Processes

**Figure 8:**
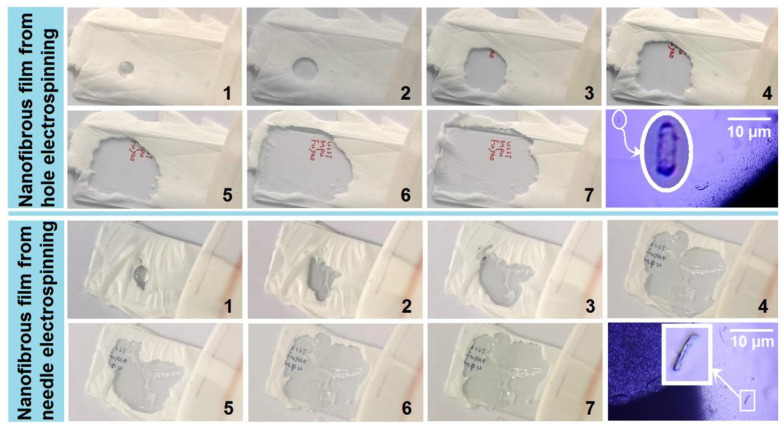
Direct comparison of nanofibrous film dissolution upon water droplet placement. Processes 1–7 occurred over ~30 s. Final blue images show dried regions where Res molecules recrystallized into microparticles (The gradually emerging abbreviation "USST MPU Fuyao" represents several collaborating units in this research).

Figure 8 presents a direct comparison of the fast-dissolution behavior of electrospun Res-loaded PVP films prepared by batch hole electrospinning and needle electrospinning. For the batch hole electrospun films, when a water droplet was placed on the nanofibrous mat, it dissolved rapidly, spreading outward to form an expanding transparent circle. The red handwritten characters “USST MPU Fuyao” became visible within approximately 30 s (Panels 1–7, upper section; see Supplementary Video S3). After drying, re-crystallized Res microparticles (~1 μm in diameter) were observed—significantly smaller than the raw Res particles. This can be attributed to the PVP molecules, which inhibit re-crystallization and crystal growth during the drying process.

A similar dissolution behavior was observed for the single-needle electrospun films. The water droplet quickly dissolved the nanofibrous mat, with the transparent area expanding to reveal the blue handwritten characters “USST MPU Fuyao” within ~30 s (Panels 1–7, bottom section). Re-crystallized Res microparticles (~1 μm) were again detected after drying, confirming the role of PVP in preventing crystal growth.

**Figure 9:**
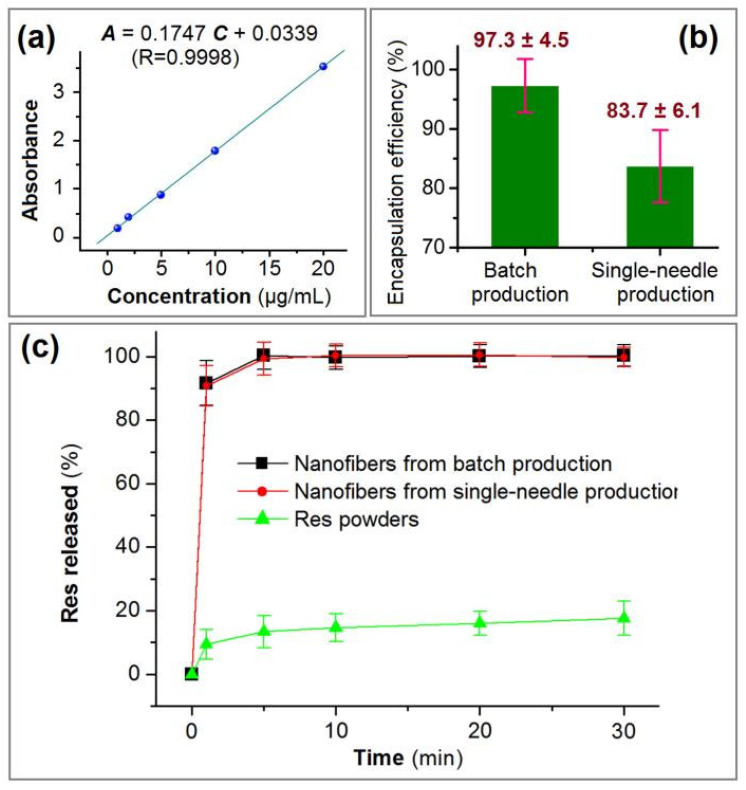
Comparison of fast-dissolution and encapsulation performance between nanofibrous films from batch production and single-fluid needle electrospinning: (**a**) standard calibration curve of Res determined by UV–vis spectrophotometry; (**b**) respective encapsulation efficiency (EE%) values of medicated nanofibers; (**c**) in vitro dissolution profiles of electrospun nanofibers and raw Res powder.

Quantitative evaluations of the fast-dissolving properties of electrospun nanofibers produced by hole electrospinning and needle electrospinning were performed using in vitro dissolution tests. Using UV–vis spectrophotometry, a standard calibration curve was obtained, and the linear regression equation is shown in Figure 9a. Within a concentration range of 1–20 µg/mL, absorbance (A) exhibited excellent linear correlation with resveratrol (Res) concentration (C, µg/mL), following the equation: A = 0.1747C + 0.0339 (R^2^ = 0.9998).

Measurements of resveratrol loading in nanofibrous films revealed 14.59 ± 0.68 mg and 12.56 ± 0.92 mg (*n* = 3) for batch-produced nanofibers and single-needle electrospun nanofibers, respectively. Thus, the drug loading (DL%) values for batch hole electrospinning and needle electrospinning were 14.59 ± 0.68% and 12.56 ± 0.92%, respectively. The theoretical drug loading for both nanofibrous types was 15.0 mg per 100.0 mg of nanofibers. Consequently, the encapsulation efficiency (EE%) values for batch hole electrospinning and needle electrospinning were 97.3 ± 4.5% and 83.7 ± 6.1%, respectively (Figure 9b). Both DL% and EE% values of batch-produced nanofibers were significantly higher than those of needle-electrospun products. Electrospinning is essentially a physical and extremely fast-drying process. The theoretical EE% should be 100%. However, Deng et al. [79] and Yang et al. [80] verified that a certain amount of drug is lost during electrospinning processes. The loss mechanism involves the escape of resveratrol molecules with the solvent into the environment. However, escaped resveratrol molecules may deposit on fluid jets and solid nanofibers, creating the false appearance that electrospun nanofibers possess 100% encapsulation efficiency. Compared with single-needle electrospinning, batch production processes provide a larger collection area to capture deposited resveratrol molecules, thereby reducing drug loss to the environment.

In vitro dissolution profiles for both types of electrospun nanofibers and raw resveratrol powders are presented in Figure 9c. The one-minute release of resveratrol from batch production, single-needle electrospinning, and raw resveratrol particles was 91.7 ± 7.1%, 90.8 ± 6.3%, and 9.4 ± 4.7%, respectively. After 5 min, the release percentages were 100.3 ± 4.3%, 99.4 ± 5.1%, and 13.4 ± 4.8%, respectively. Both batch-produced nanofibers and single-needle electrospun nanofibers released all loaded resveratrol within 5 min, demonstrating significantly superior rapid-dissolution properties compared with raw resveratrol powders, although their one-minute release kinetics differed significantly.

Res is a typical active ingredient with poor water solubility, while PVP is highly soluble in water. Their composite nanofibers dissolve rapidly when exposed to water, owing to the nanofibers’ small diameter, high porosity, large surface area, and the amorphous state of the drug. The rapid dissolution of PVP molecules enables the loaded Res molecules to be released into the water, representing a typical erosion mechanism for drug release kinetics.

### 3.5. Mechanisms of Batch Production and Its Advantages and Challenges

**Figure 10:**
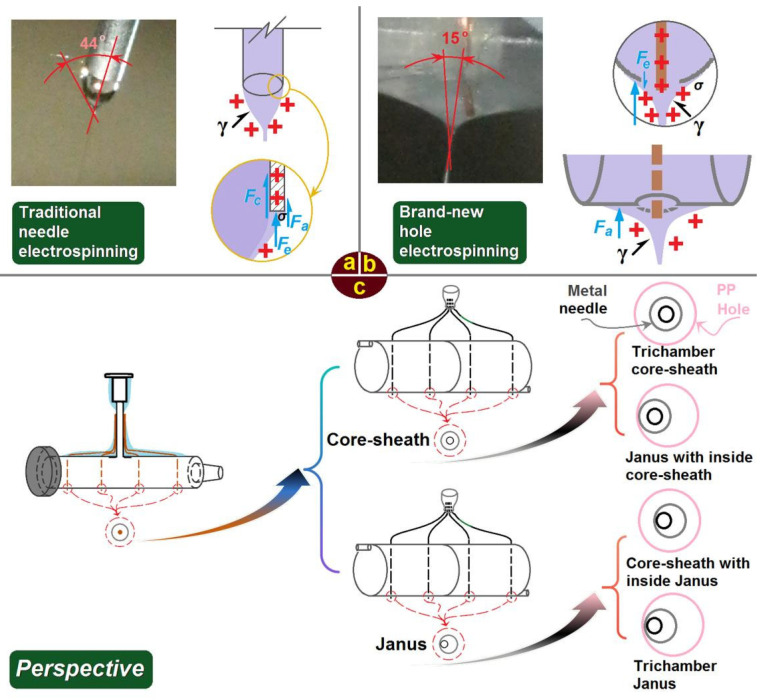
Mechanisms of (**a**) traditional needle electrospinning, (**b**) hole electrospinning, and (**c**) perspectives on batch fabrication of complex nano configurations based on the hole electrospinning concept.

Although single-fluid blending electrospinning has been broadly utilized to create various nanofibers loaded with active ingredients for functional applications, the electrohydrodynamic mechanism remains unclear. However, it is clear that the initiation of electrospinning results from a balance of various forces. As shown in Figure 10a, besides electrical forces and surface tension γ, traditional metal capillary electrospinning involves several negative factors influencing the working process. These negative forces include capillary force (Fc), adhesion force (Fa), and electrostatic force (Fe) between the working fluid and the metal. The semi-vertical angle of the Taylor cone—that is, the sharpness of the hyperboloid—depends on elastic forces and surface tension in viscoelastic fluids and typically ranges from 32° to 46° [81]. Here, the single-fluid electrospinning process had an angle of 44°, rightly falling within this range.

However, in the batch production process, the straight jet was emitted at a semi-vertical angle of 15° from the Taylor cone, falling outside the typical range, as indicated in Figure 10b. The smaller angle suggests that initiation of the working process is easier through hole electrospinning. Compared with the needle electrospinning process, there is no capillary force Fc. Meanwhile, the adhesion force Fa between the PP surface and the working fluid should be smaller than that on the metal surface. Moreover, the electrostatic force on the PP surface should be classified as electrostatic repelling force rather than the electrostatic attractive force between the metal surface and working fluid. These positive forces guiding the working fluid facilitate the easy initiation of electrospinning and provide an energy-saving process.

A direct comparison between the traditional needle electrospinning and the batch hole electrospinning processes is included in Table 2. Their products are highly similar about the physical and chemical properties. However, it is obvious that the hole electrospinning process is able to offer the following advantages: (1) batch hole electrospinning achieves a production yield 13.3 times higher (40/3 ≈ 13.3), which can be further increased simply by increasing the number of holes; (2) hole electrospinning ensures higher drug encapsulation efficiency; (3) batch production through hole electrospinning is more energy-efficient than traditional needle electrospinning; (4) the hole batch electrospinning apparatus is more compact, saving space by simplifying the fluid-driven systems; and (5) batch hole electrospinning holds great promise for the attractive mass production of complex nano configurations and nano devices in a single-step, straightforward manner.

As indicated in Figure 10c, when copper wires are replaced by metal capillaries, batch production of core-sheath nanofibers becomes possible, which is one of the most popular multiple chamber nanostructures [82,83]. When metal capillaries are arranged eccentrically, batch production of Janus nanofibers can be achieved. Moreover, when copper wires are replaced by core-sheath metal capillaries, batch production of tri-chamber nanofibers can be realized. Similarly, other tri-chamber nanofibers—such as Janus with an internal core-sheath structure, core-sheath with an internal Janus structure, and tri-chamber Janus—can be produced in a batch manner through a simple, straightforward single-step process.

Certainly, challenges remain in optimizing the batch electrospinning process, including: (1) uniform fluid distribution through multiple guide pipes to the holes of the assembled spinneret, which can be improved by increasing the fluid tank volume and the driving pressure; (2) uniform dispersion of electrical energy to the working fluids; and (3) uniformity of the resultant nanofibers across various locations of the collected nanofibrous films, which can be further addressed through the horizontal rotation of the collector.

Electrospraying, as a sister EHDA method to electrospinning, can be exploited to create nano- and microparticles in a single-step, straightforward manner. Following the progress in electrospinning, electrospraying has also been utilized to create complex configurations using multiple-fluid processes. Similarly, batch production through multiple-fluid electrospraying can be developed by referring to some most recent progresses of single-needle electrospraying [84,85]. Meanwhile, various drug fast-dissolution systems for poorly water-soluble biomolecules can be designed and developed through a combination of macromolecules and small molecules (such as β-cyclodextrin and its derivatives) and through a combination of electrospinning and electrospraying, and exhibit combinations of fibrous/particulate nano- or microcomposites [86,87].

## 4. Conclusions

A brand-new assembled hole spinneret was successfully developed for batch production of electrospun Res-loaded PVP nanofibers. Compared with the traditional metal capillary needle electrospinning, the new batch process, on one hand, can generate nanofibers with similar morphology and size distributions, similar amorphous state of the loaded active biomolecule—Res, and similar fast-dissolution profiles of the encapsulated Res molecules. On the other hand, the nanofibers batch-produced through hole electrospinning provide a larger production output, which is 13.3 times that of the traditional process. Meanwhile, the batch-produced nanofibers have a higher EE% of 97.3 ± 4.5% compared with the value of 83.7 ± 6.1% for needle electrospinning, and have an energy-saving electricity efficiency of 0.0393 W/g, which is far smaller than the 0.1247 W/g of the needle process. The microformation mechanisms of hole electrospinning reveal that it can organize the electrospinning apparatus in a more compact manner, making more effective use of the electrostatic energy while enabling large-scale creation of medicated nanofibers. A further challenge for this process is how to provide uniform dispersion of working fluids and electrostatic energy distribution to all the holes at the bottom of the spinneret. Based on the new concept of fluid guiding to the electrical field disclosed here, one of the most important and difficult challenges in nanoscience—i.e., large-scale production of complex configurations—may find its solution. Meanwhile, the hole number can be easily increased to further increase the output of nanofibers without additional pumps or power supply.

## Figures and Tables

**Table 1:** Viscosity, surface tension, and conductivity of the involved liquids.

Liquid	Viscosity (mPa·S)	Surface Tension (mN/m)	Conductivity (μS/cm)
Ethanol	0.66 ± 0.02	22.10 ± 0.03	1.00 ± 0.08
PVP solution	17.26 ± 0.35	22.92 ± 0.06	4.20 ± 0.11
PVP-Res solution	23.62 ± 0.64	22.83 ± 0.03	3.92 ± 0.13

**Table 2:** Systematic comparisons between the nanofibers from the needle and hole electrospinning.

	Process	Single-Needle Electrospinning	Hole Electrospinning
Item			
Physical property	Morphology	Linear	Linear
	Size & size distribution	725 ± 163	721 ± 140
	Porosity	68.78 ± 1.23	68.68 ±1.36
	Amorphous state	Amorphous	Amorphous
Mechanical property (tensile strength)		Highly similar mechanical behaviors	
Thermal property		Highly similar thermal responses	
Functional performances	Fast dissolution	Within 20 s	Within 20 s
	EE%	83.7 ± 6.1%	97.3 ± 4.5%
	Antioxidant activity	87.27 ± 1.10	83.65 ± 1.69
Large-scale production	Production capability	40 mL/h	40 mL/h
	Energy saving	0.0393 W/g	0.1247 W/g
	Apparatus	Simple but loose	Simple & compact
	Implementation	Easy	Easy

## Data Availability

The data supporting the findings of this manuscript are available from the corresponding authors upon reasonable.

## Supplementary Materials

The following supporting information can be downloaded at: https://www.mdpi.com/article/10.3390/polym18070795/s1, Video S1: Apparatus & parameters; Video S2: Working processes; Video S3: Fast dissoution.
